# Comparison of metabolic changes after neoadjuvant endocrine and chemotherapy in ER-positive, HER2-negative breast cancer

**DOI:** 10.1038/s41598-021-89651-0

**Published:** 2021-05-18

**Authors:** Ho Hyun Ryu, Sei Hyun Ahn, Seon Ok Kim, Jeong Eun Kim, Ji sun Kim, Jin-Hee Ahn, Kyung Hae Jung, Sung-Bae Kim, Beom Seok Ko, Jong Won Lee, Byung Ho Son, Hee Jung Shin, Hak Hee Kim, Gyung yub Gong, Hee Jeong Kim

**Affiliations:** 1 Department of Breast Surgery, University of Ulsan, College of Medicine, Asan Medical Center, 88 Olympic ro 43 gil, song pa gu, Seoul, 138-736 Korea; 2 Department of Clinical Epidemiology and Biostatistics, University of Ulsan, College of Medicine, Asan Medical Center, Seoul, Korea; 3 Department of Oncology, University of Ulsan, College of Medicine, Asan Medical Center, Seoul, Korea; 4 Department of Radiology, University of Ulsan, College of Medicine, Asan Medical Center, Seoul, Korea; 5 Department of Pathology, University of Ulsan, College of Medicine, Asan Medical Center, Seoul, Korea

**Keywords:** Cancer, Oncology

## Abstract

Survival of breast cancer patients has improved, and treatment-related changes regarding metabolic profile deterioration after neoadjuvant systemic treatment (NST) become important issues in cancer survivors. We sought to compare metabolic profile changes and the neutrophil-to-lymphocyte ratio (NLR) between patients undergoing neoadjuvant chemotherapy (NCT) and neoadjuvant endocrine therapy (NET) 3 years after the treatment. In a prospective, randomized, phase III trial which compared 24 weeks of NCT with adriamycin and cyclophosphamide followed by docetaxel and NET with goserelin and tamoxifen (NEST), 123 patients in the Asan Medical Center were retrospectively reviewed to evaluate metabolic changes, such as body mass index (BMI), blood pressure (BP), total cholesterol (TC), fasting glucose, and the NLR. The mean age of patients was 42 years. The changes in BMI, serum glucose, and TC during NST and after 3 years were significantly different between NCT and NET. The proportion of overweight + obese group and the mean BMI were significantly increased during NCT (26.6% to 37.5%, 22.84 kg/m^2^ to 23.87 kg/m^2^, p < 0.05), and these attributes found to have normalized at the 3-year follow-up. In the NET group, BMI changes were not observed (p > 0.05, all). There were no differences in changes over time among in the Hypertension group during NCT and NET (p = 0.96). The mean value of serum TC and fasting glucose significantly increased (< 0.05, both) during NCT and decreased 3 years after NCT (p < 0.05); however, no significant changes were observed in the NET group. The NLR was increased from 1.83 to 3.18 after NCT (p < 0.05) and decreased from 1.98 to 1.43 (p < 0.05) after NET. Compared with minimal metabolic effect of NET, NCT worsens metabolic profiles, which were recovered over 3 years. The NLR was increased after NCT but decreased after NET.

## Introduction

According to WHO cancer statistics, breast cancer is the most common cause of cancer-related deaths in women worldwide. With advanced treatments and the consequently improved survival, the long-term and late effects of cancer treatment are of increasing importance^1,2^. Accumulating evidence suggests that certain cancer therapies may place cancer survivors at an increased risk of worsening metabolic profiles^3–5^. Metabolic complications, such as altered glucose metabolism and increased body weight, may worsen breast cancer prognosis^6^. Plasma cholesterol levels may also influence the risk of developing breast cancer because cholesterol is a precursor of steroid hormones^7,8^.

There have been several studies on metabolic changes resulting from the treatment of breast cancer, particularly metabolic syndrome following adjuvant or NCT. Many studies have shown that metabolic biomarkers, such as waist circumference, blood pressure, and fasting levels of blood glucose, triglycerides, and high-density lipoprotein cholesterol worsen after chemotherapy^4^. Neoadjuvant chemotherapy (NCT) has been established as a standard treatment for locally advanced breast cancer, with anthracyclines and taxanes being among the most frequently used agents^9^.

Several studies have been conducted on the effects of endocrine therapy on metabolic profiles; however, they have shown conflicting results^10^. Some studies reported that tamoxifen increases insulin resistance and raises triglyceride (TG) levels^11,12^. Another study reported that tamoxifen treatment had a favorable effect on lipid metabolism by decreasing TC and LDL levels in postmenopausal women^9,13^.

However, to our knowledge, no study has compared metabolic changes after NCT and neoadjuvant endocrine therapy (NET) in young breast cancer patients who had no prior underlying condition.

Recently, there is growing evidence that cancer-related inflammatory reactions play an important role in the progression of several malignancies. For example, damage to the adaptive immune response during chronic inflammation may favor cancer cell survival and promotion of tumor growth^14–18^. The existence of an elevated peripheral neutrophil-to-lymphocyte ratio (NLR), an indicator of systemic inflammation, has been recognized as a poor prognostic factor in various cancers^19^. Neutrophilia resulting from inflammation inhibits the immune system and promote cancer progression by suppressing the cytolytic activity of immune cells, such as lymphocytes, T cells, and natural killer cells^20^. Further, some studies suggest that the NLR also functions as a metabolic marker because high NLR values may be a reliable predictive marker of insulin resistance^21^.

Prospective, randomized, phase III trial which compared 24 weeks of response of NCT and NET (NEST study), 123 patients in Asan Medical Center were retrospectively reviewed to evaluate metabolic changes such as BMI, total cholesterol(TC), fasting glucose, and NLR^22^.

The purpose of our study was to compare treatment induced changes and long term follow up in patients’ metabolic profiles and NLR ratios between NCT and NET in a series of 123 patients with locally advanced luminal-type breast cancer in young women.

## Methods/design

### Study design

The NEST study is a prospective, multicenter, randomized, parallel group, comparative phase III clinical trial. Seven centers belonging to the Korean Breast Cancer Society Group (KBCSG-012) participated in the study^22^. Our study is a substudy of the NEST study and was conducted with 123 participants evaluated and treated at the Asan Medical Center to compare the metabolic and immunologic changes between the NCT and NET arms^22^. Eligible patients included pre-menopausal women with histologically confirmed ER-positive, human epidermal growth factor receptor 2 (HER2)-negative, biopsy-proven lymph node-positive primary breast cancer^22^. Lymph node positivity was required to be histologically proven with core needle biopsy or fine needle aspiration before starting the treatment. All patients were aged between 20 and 50 years. The goal of this study was to compare the changes in metabolic profiles and NLRs following cytotoxic chemotherapy vs. GnRH agonist and tamoxifen therapy for ER-positive, HER2-negative, lymph node-positive pre-menopausal breast cancer patients.

### Procedures

Patients were randomly assigned (1:1) to receive either 3.6 mg goserelin acetate every 4 weeks with 20 mg tamoxifen daily or adriamycin and cyclophosphamide (60 mg/m^2^ adriamycin + 600 mg/m^2^ cyclophosphamide intravenously) every 3 weeks for four cycles, followed by taxol (75-mg/m^2^ docetaxel intravenously) every 3 weeks for four cycles. All patients were premedicated with oral corticosteroids dexamethasone 16 mg per day for 3 days starting 1 day prior to docetaxel administration. Treatment continued for 24 weeks, and all surgeries were performed between the 24th and 26th weeks.

The height, weight, and blood pressure (BP) of the patients were measured at the initial clinical visit. After the neoadjuvant systemic therapy (NST) method was determined, the patients underwent routine lab tests so that baseline data could be obtained, and we considered the results of the complete blood count (CBC) and chemical profile as the initial values. The patients also underwent a routine lab test as a preoperative work-up after 24 weeks of treatment. These values ​​were designated as the post-treatment values. Post-treatment body mass index (BMI in kg/m^2^) was determined based on the measurements of height and weight when patients were hospitalized for surgery. Patients continued to visit the outpatient center after surgery, and we analyzed the blood test results, height, and body weight for 3 years after the initial clinical visit. BP was measured under resting conditions (participants were seated for 5 min) using an automated BP device (FT-500, Jawon Medical Co., South Korea). A 12-h fasting blood sample was obtained for CBC with differential counts as well as a routine chemical profile including fasting glucose and total cholesterol (TC) levels. On the basis of, patients were categorized as underweight, normal weight, overweight, and obese according to the WHO classification. Initial BP was also divided into normal, elevated, and hypertension (HTN) stages 1 and 2 according to the American Heart Association (AHA) classification.

### Statistical analysis

Data were represented as the frequency and percentage for categorical variables and as the mean and standard deviation for continuous variables. The Mann–Whitney U test was used to compare continuous variables, and the chi-square or Fisher’s exact test was used to compare categorical variables. We used the linear mixed model for continuous variables and generalized estimating equations (GEE) for categorical variables to analyze the longitudinal data. The rationale behind the linear mixed model is that it can accommodate an unbalanced study design with unequally spaced repeated measures over time. Fasting glucose and NLR were analyzed after natural logarithmic transformation. All statistical analyses were performed using SAS version 9.4 (SAS Institute, Cary, NC) and R software version 3.6.0 (R Foundation for Statistical Computing). A two-sided p value < 0.05 was considered statistically significant.

### Ethical approval and consent to participate

This study protocol has been approved by the Korea Food and Drug Adminisration (KFDA) as well as institutional review board of erert trial centre, and was conducted in accordance with the Declaration Helsinki, Good Clinical Practice, and the applicable local regulatory requirements on bioethics. Written informed consent was obtained from all participants.

### Consent for publication

Authors have agreed to submit it in its current form for consideration for publication in the Journal.

## Results

### Patient and tumor characteristics

We included 64 patients in the NCT group and 59 patients in the NET group. The mean age was 41.8 ± 5.3 years in the NCT group and 42.0 ± 6.2 years in the NET group. The mean initial tumor size in the NCT group was 3.27 cm (± 1.81 cm) on ultrasonography (USG) and 3.46 cm (± 1.79 cm) on magnetic resonance imaging (MRI). In the NET group, the mean initial tumor size was 3.57 cm (± 1.80 cm) on USG and 4.16 cm (± 1.85 cm) on MRI. Other tumor characteristics, such as clinical T stage, nodal status, and estrogen and progesterone receptor (ER/PR) status, were well balanced between the two groups (Table 1) In addition, body weight groups were not statistically significantly different between both NST groups. The proportion of patients with hypertension was higher in the NET group than in the NCT group.

**Table 1:** Patient and tumor characteristics.

N (%)	NCT group (n = 64)	NET group (n = 59)	p
**Mean age ± SD (in years)**	41.80 $\pm$ 5.34	41.97 $\pm$ 6.19	0.877
**BMI**			
Underweight	4 (6.3)	3 (5.1)	0.376
Normal weight	43 (67.2)	42 (71.2)	
Overweight	16 (25)	10 (16.9)	
Obese	1 (1.6)	4 (6.8)	
HTN*			
Normal + elevated	48 (78.7)	35 (61.4)	0.040
HTN	13 (21.3)	22 (38.6)	
**Initial mean tumor size ± SD (in cm)**			
USG	3.27 $\pm 1.81$	3.57 $\pm 1.80$	0.263
MRI	3.46 $\pm$ 1.79	4.16 $\pm$ 1.85	0.026
**T stage by USG**			
T1	18 (28.1)	11 (18.6)	0.425
T2	38 (59.4)	38 (64.4)	
T3	8 (12.5)	10 (17.0)	
T4	0 (0)	0 (0)	
**Nodal status**			
N1–2	60 (93.8)	56 (94.9)	1
N3	4 (6.2)	3 (5.1)	
**Histologic grade**			
1–2	56 (87.5)	56 (94.9)	0.150
3	8 (12.5)	3 (5.1)	
**ER receptor status N (%)**			
5–6	5 (7.8)	3 (5.1)	0.719
7–8	59 (92.2)	56 (95)	
**PR receptor status**			
0–2	8 (12.5)	8 (13.6)	0.736
3–6	18 (28.1)	20 (33.8)	
7–8	38 (59.4)	31 (52.5)	

*Blood pressure were not measured in three patients from the NCT group and two patients from the NET group.

### Metabolic profiles

#### BMI

**Figure 1:**
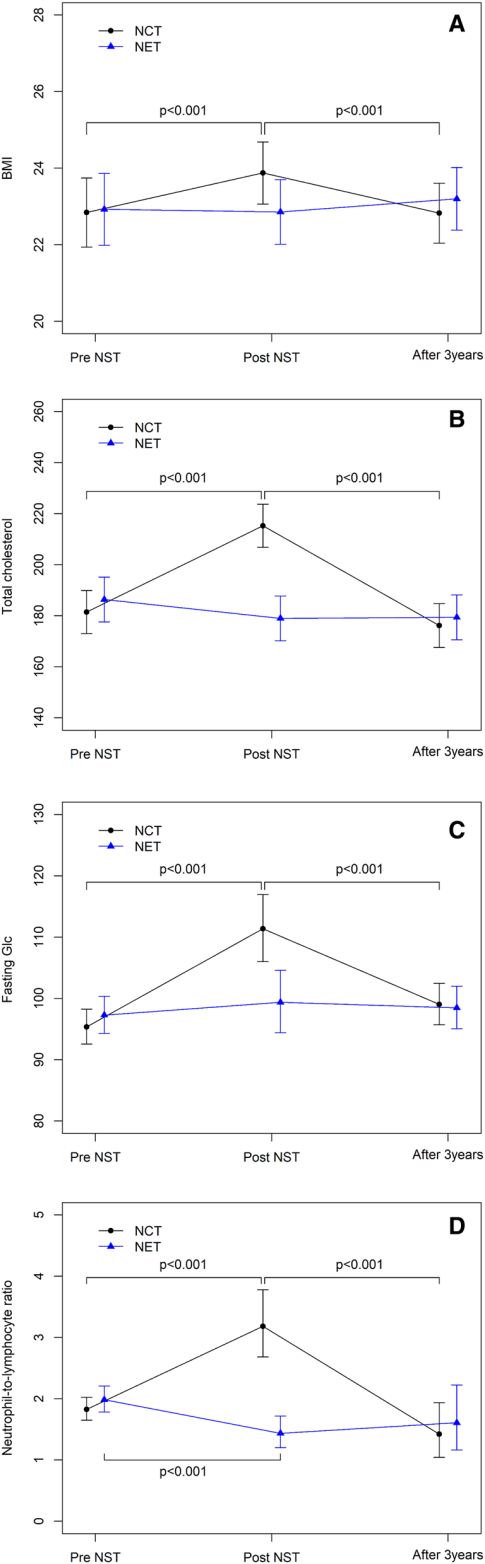
Metabolic profile and NLR changes after NST and 3 years after the initial treatment. (**A**) BMI change during NCT and NET. (**B**) Total cholesterol change during NCT and NET. (**C**) Fasting glucose change during NCT and NET. (**D**) NLR change during NCT and NET.

There was a significant difference in BMI changes between NCT and NET groups over 3 years (p < 0.05). The change in the NCT group over time was statistically significant (p < 0.05). The mean BMI before NCT was 22.84 kg/m^2^ [95% confidence interval (CI) 21.94–23.74]. After neoadjuvant treatment, BMI increased to 23.87 kg/m^2^ (95% CI 23.06–24.68) and decreased to 22.82 kg/m^2^ (95% CI 22.04–23.61) after 3 years. There was no significant difference in the NET group over this period (Table 2, Fig. 1A).

**Table 2:** Metabolic profile changes after NST and 3 years after the initial treatment.

Time	NCT group			NET group			Group p value**	Interaction between time and group*
	Means	95% CI		Means	95% CI			
**BMI**								
Pre-NST	22.84	21.94	23.74	22.92	21.98	23.86	0.901	P < 0.001
Post-NST	23.87	23.06	24.68	22.85	22.01	23.70	0.088	
3 years after treatment	22.82	22.04	23.61	23.20	22.38	24.01	0.513	
Time p value***	< 0.001			0.343				
**Total cholesterol**								
Pre-NST	181.44	172.96	189.91	186.34	177.58	195.11	0.428	P < 0.001
Post-NST	215.23	206.75	223.70	178.97	170.20	187.73	< .001	
3 years after treatment	176.15	167.58	184.72	179.38	170.56	188.19	0.605	
Time p value	< 0.001			0.177				
**Fasting Glc†**								
Pre-NST	95.36	92.55	98.26	97.28	94.29	100.36	0.365	P < 0.001
Post-NST	111.36	106.02	116.98	99.37	94.40	104.59	0.002	
3 years after treatment	99.02	95.71	102.45	98.46	95.06	101.99	0.820	
Time p value	< 0.001			0.694				

*BMI* Body mass index, *NST* Neoadjuvant systemic treatment, *NCT* Neoadjuvant chemotherapy, *NET* Neoadjuvant endocrine therapy.

*Indicates a significant difference between NCT and NET groups in changes over time.

**Comparison between two groups by specific time.

***Significance of changes over time for each group.

**Figure 2:**
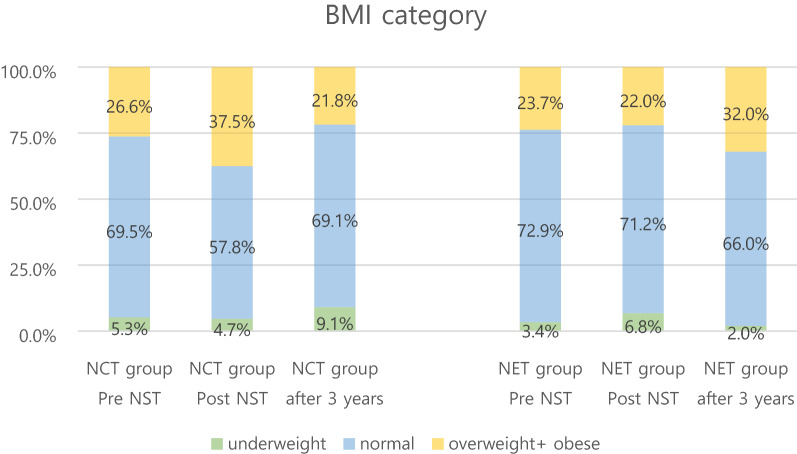
Changes in BMI category.

In the obese group, wherein the individuals had a BMI > 25 kg/m^2^ at diagnosis, the change in the NCT group over time was statistically significant (p < 0.05). There were 17 (26.6%) patients in the obese group before NCT. After neoadjuvant treatment, the number of patients in this group increased to 24 (37.5%) and then decreased to 12 (21.8%) after 3 years. There was no significant difference in the NET group in this regard. The number of obese patients before NET treatment was 14 (23.7%). Thirteen (22.0%) patients were obese after NET and 16 (32%) after 3 years (Tables 3, 4, Fig. 2).

**Table 3:** Changes in hypertension and Obese group.

Time	NCT group N (%)	NET group N (%)	Group p value**	Interaction between time and group*
**HTN stage I + II**				
Pre-NST	13 (21.3)	22 (38.6)	0.042	P = 0.964
Post-NST	20 (31.3)	30 (50.9)	0.028	
Time p value***	0.102	0.085		
**BMI ≥ 25**				
Pre-NST	17 (26.6)	14 (23.7)	0.718	P = 0.004
Post-NST	24 (37.5)	13 (22.0)	0.064	
3 years after treatment	12 (21.8)	16 (32.0)	0.241	
Time p value	0.016	0.103		

*HTN* Hypertension, *BMI* Body mass index, *NST* Neoadjuvant systemic treatment, *NCT* Neoadjuvant chemotherapy, *NET* Neoadjuvant endocrine therapy.

*Indicates a significant difference between NCT and NET groups in changes over time.

**Comparison between two groups by specific time.

***Significance of changes over time for each group.

**Table 4:** BMI category change after neoadjuvant treatment.

Group	Time	BMI category N (%)			
		Underweight	Normal	Overweight + obese	Total
NCT	Pre-NST	4 (6.3)	43 (67.2)	17 (26.6)	64
	Post-NST	3 (4.7)	37 (57.8)	24 (37.5)	64
	3 years after treatment	5 (9.1)	38 (69.1)	12 (21.8)	55
NET	Pre-NST	3 (5.1)	42 (71.2)	14 (23.7)	59
	Post-NST	4 (6.8)	42 (71.2)	13 (22.0)	59
	3 years after treatment	1 (2)	33 (66)	16 (32)	50

#### TC

There were significant differences in the TC levels between NCT and NET groups over 3 years (p < 0.05). The change in the NCT group over this period was statistically significant (p < 0.05). The mean TC before NCT was 181.44 mg/dL (95% CI 172.96–189.91). After neoadjuvant treatment, this value increased to 215.23 mg/dL (95% CI 206.75–223.70) and later decreased to 176.15 mg/dL (95% CI 167.58–184.72) after 3 years. There was no significant difference in the NET group over the time (Table 2, Fig. 1B).

#### Fasting glucose

Fasting glucose was also significantly different between NCT and NET groups after 3 years (p < 0.05). The change in the NCT group over time was statistically significant (p < 0.05). The mean fasting glucose before NCT was 95.36 mg/dL (95% CI 92.55–98.26). After neoadjuvant treatment, the value increased to 111.36 mg/dL (95% CI 106.02–116.98) and then decreased to 99.02 mg/dL (95% CI 95.71–102.45) after 3 years. The metabolic profiles showed significant differences between all time points in the NCT group (Pre-NST vs. Post-NST, Post-NST vs. 3 years p < 0.01) (Table 2, Fig. 1C). There was no significant difference in the NET group in this regard over time.

#### Blood pressure

**Figure 3:**
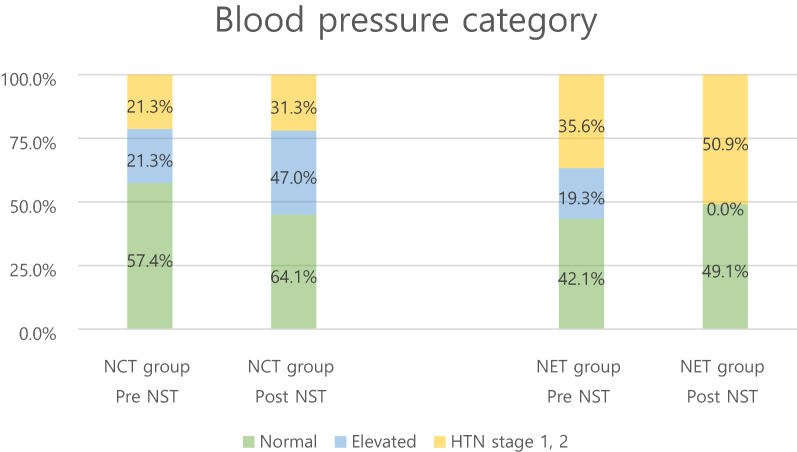
Blood pressure category changes after neoadjuvant treatment.

The proportion of HTN stage 1 and 2 patients increased from 21.3% (13) to 31.3% (20) in the NCT group and from 38.6% (22) to 50.9% (30) in the NET group (Table 5, Fig. 3). Initially, the proportion of patients with hypertension was significantly higher in the NET group than in the NCT group (Tables 1, 3). However, there were no significant differences in the changes over time between NCT and NET groups (p = 0.96) (Table 3).

**Table 5:** Blood pressure category change after neoadjuvant treatment.

Group	Time	Blood pressure category N (%)			
		Normal	Elevated	HTN stage 1, 2	Total
NCT	Pre-NST	35 (57.3)	13 (21.3)	13 (21.3)	61
	Post-NST	41 (64.1)	3 (4.7)	20 (31.3)	64
NET	Pre-NST	24 (42.1)	11 (19.3)	22 (38.6)	57
	Post-NST	29 (49.1)	0 (0)	30 (50.9)	59

#### NLR

There were significant differences in the NLR between NCT and NET groups over 3 years (p < 0.01). The change in the NCT group over time was statistically significant (p < 0.01). The mean NLR before NCT was 1.83 (95% CI 1.65–2.02). This value increased to 3.18 (95% CI 2.68–3.78) after neoadjuvant treatment and then decreased to 1.42 (95% CI 1.04–1.93) after 3 years. NLR worsening in the NCT group was caused by both neutrophilia and relative lymphopenia (Addendum 1). There was also a significant difference in the NLR in the NET group (p < 0.01). The mean NLR was 1.98 (95% CI 1.78–2.21) before NET treatment, 1.43 (95% CI 1.20–1.72) after NET treatment, and 1.61 (95% CI 1.16–2.22) after 3 years. In the NET group, the NLR changes were significantly different for all time points (Pre-NST vs. Post-NST, Post NST vs. 3 years p < 0.01), with the exception of Post-NST vs. 3-year values (Table 6, Fig. 1D).

**Table 6:** NLR changes after neoadjuvant systemic treatment.

Time	NCT group			NET group			Group p value**	Interaction between time and group*
	Means	95% CI		Means	95% CI			
NLR								
Pre-NST	1.83	1.65	2.02	1.98	1.78	2.21	0.273	P < 0.001
Post-NST	3.18	2.68	3.78	1.43	1.20	1.72	< 0.001	
3 years after treatment	1.42	1.04	1.93	1.61	1.16	2.22	0.579	
Time p value***	< 0.001			0.003				

*NLR* Neutrophil-to-lymphocyte ratio, *NST* Neoadjuvant systemic treatment, *NCT* Neoadjuvant chemotherapy, *NET* Neoadjuvant endocrine therapy.

*Indicates a significant difference between NCT and NET groups in changes over time.

**Comparison between two groups by specific time.

***Significance of changes over time for each group.

## Discussion

To our knowledge, this is the first study to demonstrate a relationship between metabolic profiles and NLR variation during neoadjuvant treatment in locally advanced luminal-type breast cancer in young women. This study has shown that NCT worsens metabolic profiles with unfavorable changes in BMI, total cholesterol, and fasting glucose, which recover over 3 years. In the NET group, there were no significant changes in BMI, TC, and fasting glucose. Conversely, NCT increased the NLR, whereas NET decreased the NLR, which recovered over 3 years.

Weight gain during chemotherapy for breast cancer has been well studied, but the reason for this weight gain is not fully understood^23^. Low physical activity, reduced resting metabolic rate, reduced thermogenesis, chemotherapy induced edema and unreduced dietary intake during chemotherapy^24^ are all possible reasons for weight gain during chemotherapy; however, no conclusive data exist to support any of these mechanisms^25^. Many studies have shown that metabolic biomarkers worsen after chemotherapy^4^. Explanations of potential mechanisms exist to explain the metabolic deterioration after chemotherapy-associated weight gain itself. Some hypotheses suggest that chemotherapy causes endothelial damage which leads to some cytokine alterations, which have an effect on the development of metabolic profile deterioration^26,27^. As there was a significant increase in BMI after NST in the NCT group, we reclassified the patients based on their BMI to determine the characteristics of those who were initially in the normal BMI group but became overweight after treatment. Eight patients met these criteria, but the sample size was too small to use statistical techniques and showed no special features. Also changes in self-perceived health and mental attitudes with weight gain during NCT, as well as metabolic profiles, could be considered one of the interesting topics. And this is carried out as our upcoming research.

Many studies have been conducted on the effects of endocrine therapy on metabolic profiles, and results are inconsistent. Some studies reported that tamoxifen had an adverse effect on metabolic profiles^11,12^; conversely, another study reported that tamoxifen had a favorable effect on lipid metabolism^9,13^ The mechanisms by which tamoxifen affects lipids and lipoproteins are unknown. However, some researchers have suggested that tamoxifen’s estrogenic effects on the hepatic lipoprotein metabolism, which lower LDL levels and increase the synthesis of apolipoprotein A-I, result in high concentrations of high-density lipoprotein (HDL)^13^. Our study also showed a decrease in TC during NET; however, the changes were not statistically significant.

Elevated NLR after NCT was due to relative neutrophilia, which occurs as a part of the systemic inflammatory response triggered by cancer^28–31^. Recent studies have indicated that metabolic profiles are associated with many chronic inflammation risk factors and that the NLR could be a predictive marker for developing metabolic syndrome^32,33^. It has also been suggested that increased NLR is associated with type 2 diabetes mellitus and increased risk for cardiovascular events^34^. In our study, the NLR was increased after NCT and decreased after NET, which were well corelated with other metabolic profiles.

High NLR is also known to be associated with an adverse overall survival (OS) and disease free survival (DFS) in patients with breast cancer^35–38^. . A few studies analyzing the NLR changes after cancer treatment have been conducted; these studies showed that the NLR after cancer treatment has its own significancy as well as pretreatment NLR^35^. There are many studies on pretreatment NLR and its significance, but there is also accumulating evidence suggesting that the NLR after treatment is associated with breast cancer-free survival with late recurrence^39^. In our study, the NLR was worsened by NCT and improved by NET. These NLR changes should be further investigated with survival analysis.

This study has a few limitations. The number of patients was small. Not all patients received the same tests; thus, there were intermediate blank data for the NLR or BMI. Although this gap in data was overcome by statistical techniques, with more patients, it would have been possible to draw more meaningful and reliable conclusions. Another limitation is that certain tests may be included in the metabolic profile, such as HDL and LDL cholesterol and TG, which were not included in the study. Further investigations should be performed to overcome these limitations.

Despite these limitations, the strength of this study is that it was performed using data collected during neoadjuvant treatment for breast cancer. Many studies have been conducted in the adjuvant setting, but few have compared NCT and NET among locally advanced breast cancer patients. Moreover, this study evaluated metabolic changes for young women who had fewer comorbidities than older women; thus, any changes observed in their metabolic profiles are more likely to be a result of the treatment itself. Finally, we herein evaluated metabolic changes not only during NST but also chronologically at 3 years after treatment. To our knowledge, no study has compared the effects of the two neoadjuvant treatments directly in detail or analyzed the long-term data of up to 3 years after the start of the treatment.

## Conclusion

NCT worsens metabolic profile parameters, such as BMI, TG, and fasting glucose, which are then recovered over 3 years. In the NET group, however, there were no significant changes in BMI, fasting glucose, and TC. BP was unaffected in both groups, whereas NLR was increased after NCT but decreased after NET.

## Supplementary Information

Supplementary Information.

## Data Availability

The data supporting the conclusions of this article are included within the article. Any queries regarding these data may be directed to the corresponding author.
