# Supplementary material for: Comparison of metabolic changes after neoadjuvant endocrine and chemotherapy in ER-positive, HER2-negative breast cancer
Source: Sci Rep. 2021 May 18;11:10510. doi: 10.1038/s41598-021-89651-0 (PMC8131718; doi:10.1038/s41598-021-89651-0)
Supplement: Supplementary file 1 — Supplementary Information. [file 41598_2021_89651_MOESM1_ESM.docx]

Addendum 1. Neutrophil (%) and lymphocyte (%) changes during NST and 3 years after the initial treatment

|  | Time | NCT group | | | NET group | | | Group p value** | Interaction between  Time and Group* |
| --- | --- | --- | --- | --- | --- | --- | --- | --- | --- |
|  |  | means | 95% CI | | means | 95% CI | |  |  |
| Neutrophil  (%) | Pre-NST | 58.652 | 56.336 | 60.968 | 60.593 | 58.181 | 63.005 | 0.2526 | <.0001 |
|  | Post-NST | 68.153 | 64.673 | 71.633 | 52.871 | 49.247 | 56.495 | <.0001 |  |
|  | 3 years after treatment | 52.599 | 46.875 | 58.323 | 54.035 | 48.017 | 60.054 | 0.7284 |  |
|  | Time p value*** | <.0001 | | | 0.0006 | | |  |  |
| Lymphocyte  (%) | Pre-NST | 32.333 | 30.395 | 34.271 | 30.854 | 28.836 | 32.873 | 0.2976 | <.0001 |
|  | Post-NST | 21.895 | 19.509 | 24.281 | 37.453 | 34.967 | 39.938 | <.0001 |  |
|  | 3 years after treatment | 37.559 | 32.211 | 42.907 | 34.829 | 29.207 | 40.452 | 0.481 |  |
|  | Time p value | <.0001 | | | <.0001 | | |  |  |

* Indicates a significant difference between NCT and NET groups in changes over time

** Comparison between two groups by specific time

*** Significance of changes over time for each group

NST: Neoadjuvant systemic treatment; NCT: Neoadjuvant chemotherapy; NET: Neoadjuvant endocrine therapy
